# Dietary Behaviors and the Living Environment Can Explain Residual Obesity Risk

**DOI:** 10.3390/nu17091413

**Published:** 2025-04-23

**Authors:** Demosthenes Panagiotakos

**Affiliations:** Department of Nutrition and Dietetics, School of Health Sciences and Education, Harokopio University, 176 76 Athens, Greece; dbpanag@hua.gr

## 1. Introduction

Despite substantial advancements and extensive funding in obesity research—spanning the development of novel pharmacological and non-pharmacological treatments, as well as numerous public health initiatives—the global prevalence of obesity continues to escalate at an alarming rate. 

Obesity is a complex condition influenced by both genetic and environmental factors [1]. It has been suggested that genetic predisposition accounts for approximately 40–70% of an individual’s risk of developing obesity [2,3,4]. While genetics play a critical role in obesity risk, lifestyle factors such as diet and physical activity are also well recognized for their significant impact on an individual’s body weight [5,6,7].

However, the persistent increase in obesity rates suggests that there are key factors that have been overlooked or not even considered in current obesity research, increasing the residual risk. This residual obesity risk refers to the influence of certain behavioral, psychological, and socio-environmental determinants that are not accounted for in conventional models. Factors such as stress, emotional eating, sleep deprivation, food insecurity, and cultural norms surrounding diet and physical activity can significantly impact an individual’s ability to maintain a healthy weight, even when genetic predisposition and conventional risk factors are considered. Additionally, socioeconomic status, exposure to obesogenic environments, and marketing of unhealthy foods contribute to persistent weight-related disparities.

This editorial explores how dietary behaviors, including meal timing, portion control, food choices, and eating patterns influenced by emotional and environmental cues, may contribute to residual obesity risk. It also examines why traditional obesity interventions—typically focused on calorie restriction, exercise, and behavioral modification—often fail to address these deeper, underlying factors. By recognizing the broader influences on eating behaviors, we can develop more effective and sustainable strategies for obesity prevention and management.

## 2. Advances in Obesity Research and Their Limitations

Over the past few decades, significant strides have been made in understanding the pathophysiology of obesity. Advances in metabolic research have identified key pathways involved in energy regulation, appetite control, and fat storage. Genetic variations can affect metabolism, appetite regulation, fat storage, and energy expenditure, making some individuals more susceptible to weight gain. Studies have identified multiple genes, such as FTO (i.e., fat mass and obesity-associated gene) and melanocortin-4 receptor gene (MC4R), that play a role in obesity by influencing hunger, satiety, and fat accumulation [3,4]. 

Recent research suggests that gut microbiota composition may play a pivotal role in residual obesity risk. The gut microbiome influences energy extraction from food, metabolic rate, and inflammation, factors not typically measured in standard obesity studies. Dysbiosis, or an imbalance in gut microbiota, has been linked to increased adiposity and altered energy metabolism, independent of caloric intake. Furthermore, epigenetic modifications induced by early-life nutrition and dietary exposures may predispose individuals to obesity later in life. Maternal diet, childhood eating patterns, and intergenerational influences contribute to a metabolic phenotype that traditional interventions may fail to reverse [8,9,10].

Moreover, pharmaceutical innovations, such as GLP-1 receptor agonists (e.g., semaglutide and tirzepatide), have emerged as promising treatments for obesity, demonstrating efficacy in reducing body weight. Additionally, public health initiatives have promoted dietary guidelines aimed at reducing calorie intake and improving nutrient quality [11].

However, despite these advances, obesity rates continue to climb. This paradox suggests that conventional interventions, focused on diet composition, caloric restriction, and pharmacotherapy, do not fully address the complexity of obesity. The concept of residual obesity risk acknowledges that many non-traditional factors, particularly those linked to dietary behaviors, remain unrecognized in standard epidemiological frameworks. This residual obesity risk encompasses factors that are not typically included in obesity research models but nonetheless exert a significant influence on weight regulation. These include eating behaviors and patterns, i.e., irregular meal timing, frequent snacking, and nighttime eating, which have been associated with obesity risk beyond total caloric intake, emotional eating, stress-induced overeating, and hedonic eating that contribute to weight gain independent of metabolic factors, disrupted circadian rhythms, and late-night food consumption and negatively impact metabolic homeostasis [12].

## 3. Why Traditional Approaches Fall Short

Conventional dietary interventions for obesity often focus on nutrient composition and calorie reduction. However, these approaches do not fully address the behavioral and environmental contributors to obesity risk. It is worth noting that most intervention studies have been meticulously and thoughtfully designed to achieve their objectives and funding from national and international organizations; however, their long-term sustainability remains uncertain. Key shortcomings include failure to address certain behavioral triggers. Many weight loss programs neglect the role of emotional eating and stress-induced dietary behaviors. Cognitive and behavioral interventions, such as mindfulness-based eating strategies, are underutilized in obesity management. Moreover, public health recommendations often assume equal access to healthy foods, ignoring disparities in food availability and affordability. Stress, work schedules, and cultural factors shape dietary behaviors in ways that standard interventions fail to consider [13,14].

The food environment also plays a significant role in shaping dietary behaviors and obesity risk. The availability, affordability, and accessibility of healthy versus unhealthy foods can greatly influence an individual’s eating patterns and overall caloric intake. In many urban settings, highly processed, energy-dense foods are more convenient and cost-effective than fresh, nutrient-rich options, making it challenging for individuals, especially those from lower socioeconomic backgrounds, to maintain a balanced diet. Additionally, aggressive marketing strategies by the food industry, particularly targeting children and low-income populations, further contribute to unhealthy eating habits. Factors such as portion sizes, food deserts, workplace and school meal options, and cultural norms around eating also shape long-term dietary behaviors. As a result, even individuals who are aware of healthy eating principles may struggle to make healthier choices in environments that continuously promote overconsumption of unhealthy foods. Addressing these systemic influences through policy changes, urban planning, and educational initiatives is essential for reducing obesity risk at the population level [15,16,17].

## 4. Strategies to Mitigate Residual Obesity Risk

To effectively combat obesity, interventions must integrate a broader understanding of dietary behaviors and their interactions. Urban planning initiatives can increase access to healthy food options in underserved communities. School-based nutritional programs can instill lifelong healthy eating habits in children. Behavioral interventions should be incorporated into obesity management strategies. Dietary modifications should extend beyond calorie reduction. Emphasizing whole foods, fiber-rich diets, and gut microbiome-friendly nutrition can promote better long-term outcomes. Public health and policy initiatives must also be strengthened. Food labeling regulations should be improved to enhance consumer awareness of food quality. Urban planning initiatives should focus on increasing access to healthy food options in underserved communities. School-based nutritional programs should aim to instill lifelong healthy eating habits in children.

## 5. Conclusions

The persistence of obesity despite advances in current research highlights the significance of residual obesity risk. Dietary behaviors, shaped by social and environmental factors, play a crucial role in obesity that is not adequately captured in traditional studies. Addressing residual obesity risk requires a paradigm shift in obesity prevention and management, incorporating behavioral interventions, dietary quality improvements, and systemic policy changes. A holistic approach that integrates these factors is essential to curbing the global obesity epidemic and improving long-term health outcomes.
